# Influence of Estrus on the Milk Characteristics and Mid-Infrared Spectra of Dairy Cows

**DOI:** 10.3390/ani11051200

**Published:** 2021-04-22

**Authors:** Chao Du, Liangkang Nan, Chunfang Li, Ahmed Sabek, Haitong Wang, Xuelu Luo, Jundong Su, Guohua Hua, Yabing Ma, Shujun Zhang

**Affiliations:** 1 Key Lab of Agricultural Animal Genetics, Breeding and Reproduction of Ministry of Education, Huazhong Agricultural University, Wuhan 430070, China; dc1992hml@163.com (C.D.); 15827557518@163.com (L.N.); ahmedsabek1987@gmail.com (A.S.); wanghaitong0411@163.com (H.W.); lxl775282323@163.com (X.L.); 13430376634@163.com (J.S.); huaguohua09@gmail.com (G.H.); 2 Hebei Livestock Breeding Station, Shijiazhuang 050000, China; chunfangli0521@126.com (C.L.); dhimyb@163.com (Y.M.); 3 Department of Veterinary Hygiene and Management, Faculty of Veterinary Medicine, Benha University, Moshtohor 13736, Egypt

**Keywords:** dairy cow, estrus, milk production traits, mid-infrared spectrum

## Abstract

**Simple Summary:**

Some studies have confirmed the variation in milk profiles when dairy cows show estrus. However, only a few milk components, such as fat, protein, and lactose, have been investigated so far, and thus any changes in the many other parts of milk’s composition due to estrus are unknown. Milk mid-infrared (MIR) spectra consist of wavenumbers, which provide insight into the chemical composition of milk. The MIR spectrum reflects the global composition of milk, but this information is currently underused. In this study, we considered MIR wavenumbers as traits, and directly studied the spectral information as a way to study the estrus of dairy cows linked to milk composition. This research provides a deeper understanding of the milk MIR spectrum and may lead to new approaches for estrus detection in dairy cows from routine milk analysis, thereby guiding an opportune insemination time.

**Abstract:**

Milk produced by dairy cows is a complex combination of many components. However, at present, changes in only a few milk components (e.g., fat, protein, and lactose) during the estrus cycle in dairy cows have been documented. Mid-infrared (MIR) spectroscopy is a worldwide method routinely used for milk analysis, as MIR spectra reflect the global composition of milk. Therefore, this study aimed to investigate the changes in milk MIR spectra and milk production traits (fat, protein, lactose, urea, total solids (TS), and solid not fat (SnF)) due to estrus. Cows that were successfully inseminated, leading to conception, were included. Cows confirmed to be pregnant were considered to be in estrus at the day of insemination (day 0). A general linear mixed model, which included the random effect of cows, the fixed classification effects of parity number, days in relation to estrus, as well as the interaction between parity number and days in relation to estrus, was applied to investigate the changes in milk production traits and 1060 milk infrared wavenumbers, ranging from 925 to 5011 cm^−1^, of 371 records from 162 Holstein cows on the days before (day −3, day −2, and day −1) and on the day of estrus (day 0). The days in relation to estrus had a significant effect on fat, protein, urea, TS, and SnF, whose contents increased from day −3 to day 0. Lactose did not seem to be significantly influenced by the occurrence of estrus. The days in relation to estrus had significant effects on the majority of the wavenumbers. Besides, we found that some of the wavenumbers in the water absorption regions were significantly changed on the days before and on the day of estrus. This suggests that these wavenumbers may contain useful information. In conclusion, the changes in the milk composition due to estrus can be observed through the analysis of the milk MIR spectrum. Further analyses are warranted to more deeply explore the potential use of milk MIR spectra in the detection of estrus.

## 1. Introduction

Good reproductive management has an immediate effect on milk production and the economic success in dairy cattle farms. Efficient estrus detection and subsequently an opportune insemination time are fundamental components of a successful reproductive management program [1]. Estrus is typically detected according to some specific behavioral signs. Standing to be mounted by fellows is often considered as the most meaningful factor for estrus detection. Other behavioral features characterizing estrus include, for instance, restlessness, sniffing the vulva of another cow, and licking [2]. The traditional methods of visual estrus detection are time-consuming, ineffective, and increase the workload of farm staff. Nowadays, several innovative and automated tools have been developed to detect estrus, such as pressure-sensing devices [3], body temperature detectors [4], neck-mounted collars to detect physical activity [5], and pedometers [6]. However, these technologies may require potentially burdensome investments in equipment and management.

Some studies have confirmed the variation in milk profiles when dairy cows show estrus. However, milk is a complex combination of at least 10,000 different biomolecules [7]. To our knowledge, at present, only a few milk components have been investigated. For example, Toledo-Alvarado et al. [8] reported that 24 milk traits, including lactose, fat, protein, casein, urea, the fat:protein ratio (F:P), the freezing-point depression (FPD), the homogenization index (HI), and some specific fatty acids, were significantly affected by estrus phases. Specifically, fat increased from the diestrus to estrus phase, whereas protein concomitantly decreased. Specific fatty acids, such as myristic acid and palmitic acid, decreased from proestrus to estrus with a concomitant increase in stearic acid and oleic acid. Zebari et al. [9] found that the concentration of acetic acid, valeric acid, caproic acid, and myristoleic were greater in milk on the day of estrus compared to the diestrus period, whereas the milk arachidonic acid concentration was greater in the diestrus period compared to the day of estrus. Zhao et al. [10] showed that inactive-ovary cows compared with normal-estrus cows at 70 days postpartum had an increase in the milk whey of six different metabolites, including succinate, creatine phosphate, glycine, myo-inositol, glycolate, and orotate, and a decrease in the milk whey of seven metabolites, including alanine, creatinine, o-phosphorylcholine, lactose, taurine, galactose, and glucose-1-phosphate. These means that the changes of a large part of other milk composition elements due to estrus is unknown.

Mid-infrared (MIR) spectroscopy is a fast and cost-effective method globally used to routinely assess the milk composition, such as the protein [11], fat, casein, lactose, total solid (TS), and urea [12]. In addition, MIR spectra also have the potential to determine many other milk components, such as the lactoferrin content [13], fatty acids profiles [14], free amino acid [15], acetone, β-hydroxybutyrate (BHB), citric acid [16], mineral composition [17], and protein fraction compositions [18], although the predictive accuracy for some milk components need further improvement. Milk spectra consist of wavenumbers, which provide insight in the chemical composition of milk. The MIR spectrum reflects the global composition of milk, but this information is currently underused. Therefore, it could be interesting to consider MIR wavenumbers as traits, and directly study the spectral information as a way to study the estrus of dairy cows linked to the milk composition. The present study, therefore, was conducted to investigate the changes in MIR spectral wavenumbers and milk characteristics due to estrus. We hypothesized that estrus had a significant effect on some of the milk composition traits and MIR spectral wavenumbers. This research provides a deeper understanding of the milk MIR spectra, and may lead to new approaches for estrus detection directly from routine milk analysis, thereby guiding the opportune insemination time of the dairy cows.

## 2. Materials and Methods

### 2.1. Animal Management and Milk Samples 

Lactating Holstein dairy cows (*n* = 309) with 1 to 5 parities (first lactation, *n* = 73; second lactation, *n* = 76; third lactation, *n* = 50; fourth lactation, *n* = 85; and fifth lactation, *n* = 25) on a commercial dairy farm (longitude 115°47′ E, latitude 37°20′ N) in Hebei province, China, were used in this study. Cows were kept in free stall barns with cubicles equipped with a concrete solid floor and had free access to the feeding area. An ad libitum total mixed ration (TMR), consisting of corn silage, grass silage, concentrate, straw, and additives, were provided. Fresh TMR was supplied 3 times per day at 08:00, 16:00, and 24:00. The TMR was consistent during the experiment. Water was available continuously and ad libitum via troughs placed in the feeding area. The cows were milked three times daily at 07:00, 15:00, and 23:00 in a milk carousel with 80 milk stalls. The average daily milk yield of the analyzed cows was 35 kg/day.

During the experimental period (between November and December 2019), cows at 15–21 days post-partum were synchronized with a Presynch-Ovsynch protocol (prostaglandin F_2α_ (PGF_2α_), 14 days later PGF_2α_, 7 days later gonadotropin-releasing hormone (GnRH), 7 days later PGF_2α_, 3 days later GnRH, 7 days later GnRH, 7 days later PGF_2α_, 24 h later PGF_2α_, 32 h later GnRH, and 14 h later timed artificial insemination (TAI)). Day of TAI was designated day 0. Pregnancy diagnosis was performed by an experienced veterinarian using both rectal palpation and ultrasound examination 5 weeks after TAI. Milk samples were collected from day −3 to day 0. The days in milk of those cows during collection ranged from 59 to 68 days. During the morning milking, about 40 mL of milk was sampled. Samples were immediately added with preservative (2-bromo-2-nitropropan-1, 3-diol) and kept at 4 °C until analysis. The animal handling procedures and all experimental protocols were approved by the Scientific Ethic Committee of Huazhong Agricultural University (permit number HZAUCA-2019-005).

### 2.2. Milk Characteristics and MIR Spectral Data

Milk samples were sent to the Hebei Livestock Breeding Station within 24 h of collection to be analyzed for fat, protein, lactose, urea, total solids (TS), and solid not fat (SnF) using CombiFoss FT + (Foss, Hillerød, Denmark), and the corresponding spectra were obtained for this study. Furthermore, the CombiFoss FT+ device was calibrated at regular times using standard samples, and the milk MIR spectra were standardized according to the manufacturer’s instructions. A recorded spectrum includes 1060 data points, with each point representing the absorption of infrared light through the milk sample at a particular wavenumber in the 5011 to 925 cm^−1^ region. To ensure that cows showed true estrus, only data from cows with insemination leading to conception (*n* = 162) were included in the analysis of the changes in milk components and mid-infrared spectra on the days before and on the day of estrus. Cows confirming to be pregnant were considered to be in estrus at the day of insemination.

### 2.3. Statistical Analysis

A generalized linear mixed model was used to test the changes in the milk components and mid-infrared spectra. Data were analyzed using PROC MIXED of SAS (version 9.4; SAS Institute Inc., Cary, NC). For each trait, the model was defined as follows: y_ijk_ = μ + parity_i_ + day_j_ + parity_i_ × day_j_ + cow_k_ + e_ijk_, where y_ijk_ is the response on the trait (fat, protein, lactose, urea, TS, SnF, and absorbance value of the 1060 infrared points); μ is the general mean; parity_i_ is the fixed effect of the parity number i (i = 1, 2, 3, 4, and 5); day_j_ is the fixed effect of day j (j = −3, −2, −1, and 0); parity_i_ × day_j_ is the effect of the interaction between the parity i and the day j; cow_k_ is the random effect of cows accounting for the repeated measurements; and e_ijk_ is the random residual error. Bonfferoni correction was used to adjust for multiple testing of the differences between the effect levels, and *p* < 0.05 was considered significant.

## 3. Results

### 3.1. Descriptive Statistics

Descriptive statistics of the milk components are reported in Table 1. The overall mean fat, protein, and lactose was 4.06 ± 1.04%, 3.68 ± 0.51%, and 5.21 ± 0.26%, respectively. Fat showed large variability (coefficient of variation (CV) = 25.62%) relative to protein (CV = 13.86%) and lactose (CV = 4.99%). Urea averaged 18.81 ± 5.86 mg/100 g (CV = 31.15%), and ranged from 5.80 to 33.90 mg/100 g. The average total solid (TS) was 13.81 ± 1.34% (CV = 9.70%), with values ranging from 7.78 to 17.60%. Solid not fat (SnF) averaged 9.62 ± 0.57%, with a coefficient of variation of 5.93%.

**Figure 1:**
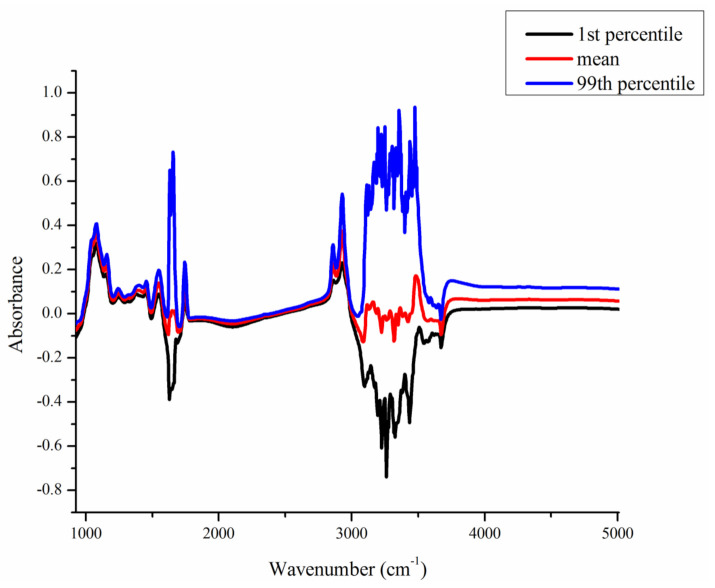
The mean absorbance for 1060 infrared wavenumbers, and the corresponding 1st percentile and 99th percentile, based on records of 371 milk samples.

The mean, 1st percentile, and 99th percentile of the 1060 individual spectral points are shown in Figure 1. In the present study, we characterized the milk spectra into 5 regions, namely, from wavenumbers 925 to 1608 cm^−1^ (the first region), from wavenumbers 1612 to 1678 cm^−1^ (the second region), from wavenumbers 1682 to 3055 cm^−1^ (the third region), from wavenumbers 3059 to 3661 cm^−1^ (the fourth region), and from wavenumbers 3665 to 5011 cm^−1^ (the last region). There were two regions, including wavenumbers between 1612 and 1678 cm^−1^ (the second region) and between 3059 and 3661 cm^−1^ (the fourth region), that showed larger variation than the others. These wavenumbers represent the absorption peaks of water. The first region spans wavenumbers 925 to 1608 cm^−1^. The absorbance value of wavenumber 925 cm^−1^ was −0.07 and increased rapidly to the value of 0.37 at wavenumber 1076 cm^−1^, and then decreased to the value of −0.08, observed at wavenumber 1608 cm^−1^. The third relevant region spans wavenumbers 1682 to 3055 cm^−1^. The absorbance value gradually increased from wavenumbers 1682 to 2839 cm^−1^, corresponding to values from −0.04 to 0.13. After wavenumber 2843 cm^−1^ (0.15), the absorbance values sharply increased to 0.38 at wavenumbers 2928 cm^−1^, and then decreased to −0.08 at wavenumber 3055 cm^−1^. The last region includes 350 spectral points in the interval from wavenumbers 3665 to 5011 cm^−1^. The absorbance value started at −0.05 at wavenumber 3665 cm^−1^, increased to 0.06 at wavenumber 3734 cm^−1^, and then the absorbance values were relatively constant, varying little from 3734 cm^−1^ to 5011 cm^−1^.

### 3.2. Changes in the Milk Components and MIR Spectra

**Figure 2:**
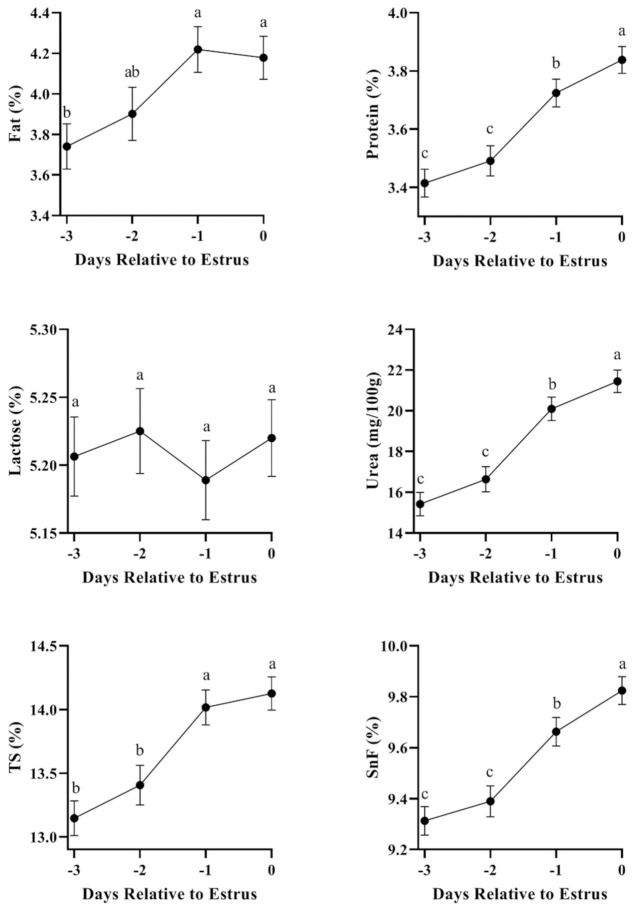
Changes in fat, protein, lactose, urea, total solid (TS), and solid not fat (SnF) from day -3 to day 0. Results are given as the LSQ means ± SE. ^a,b,c^ Means with different superscripts differ significantly at *p* < 0.05.

Fat, protein, urea, TS, and SnF were significantly influenced by the days in relation to estrus. The fat increased from day −3 to day −1, and then slightly decreased on day 0, and its content was significantly higher on day −1 and day 0 compared to day −3. The changes in protein, urea, TS, and SnF showed a similar pattern, which increased from day −3 to the day of estrus. The protein, urea, and SnF on the day of estrus were significantly higher compared to the other three days, whereas the TS was significantly higher on day −1 and day 0 compared to day −3 and day −2. No significant differences in lactose were found between the day of estrus and the other three days, and its content in milk showed an erratic pattern (Figure 2).

**Figure 3:**
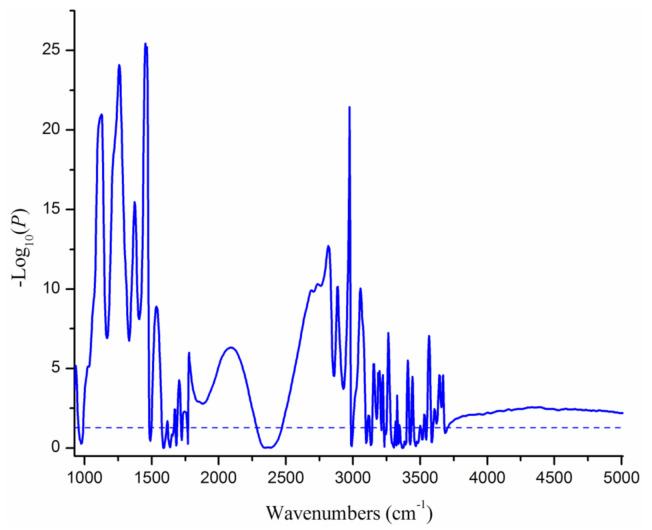
The significance of the effect of the days in relation to estrus on milk infrared wavenumbers. The horizontal line indicates a threshold at −log_10_(*p*) of 1.3.

Figure 3 shows the −log_10_(*p*) value of the days in relation to estrus effect on the 1060 individual spectral points. The days in relation to estrus had significant effects on 891 spectral points, most of which belonging to the first (925 to 1608 cm^−1^), third (1682 to 3055 cm^−1^), and last regions (3665 to 5011 cm^−1^). Besides, some wavenumbers, such as from 3059 to 3090 cm^−1^ and from 3144 to 3202 cm^−1^, which are in the water absorption regions, are also significantly affected by estrus. Especially, some spectral points were extremely changed on the days before and on the day of estrus. The days in relation to estrus had no significant effect on 169 spectral points; of these, 80 spectral points belong to the water absorption regions.

## 4. Discussion

In this study, we investigated the changes in milk characteristics on the days before and on the day of estrus. The days in relation to estrus had significant effects on fat, protein, urea, TS, and SnF, but not lactose. The milk fat content increased from 3 days before estrus to the day of estrus. An increment in the fat content during estrus also has been described in other studies [8]. Protein showed a similar tendency with fat from 3 days before estrus to the day of estrus. In accordance with a previous study [19], the authors reported that the protein percentage was significantly affected by the stage of the estrus cycle, and an increase in protein was observed during estrus. Conversely, Toledo-Alvarado et al. [8] showed an opposite pattern, in that protein decreased gradually from 3 days before estrus to the estrus day. Akdag et al. [20] compared the difference of lactose between the estrus period and 3 days after estrus, and found that estrus had no significant effect on lactose, which is in agreement with the present study. Moroni et al. [19] also indicated that the milk lactose percentage was not affected by the stage of the estrous cycle. However, Toledo-Alvarado et al. [8] reported that the estrus phase significantly influenced lactose, which gradually increased from 3 d before estrus to the day of estrus. The urea of milk increased from 3 days before estrus to the day of estrus. Toledo-Alvarado et al. [8] reported that milk urea showed an erratic pattern during an estrus cycle. These discrepancies between the present study and the study of Toledo-Alvarado et al. [8] could be due to several reasons, such as total records, number of records for per animal, sample size, breeds, and the statistical and analytical model. However, the most likely reason may be the difference in the definition of estrus. In the study of Toledo-Alvarado et al. [8], the authors considered the day of insemination to be estrus without regard for the conception outcome. In the present study, only cows with insemination leading to conception were considered to be in estrus at the day of insemination. Besides, the current study also indicated the significant changes in TS and SnF on the days before and on the day of estrus, both of which increased from 3 days before estrus to the estrus day. The changes in milk characteristics around estrus indicate their potential as a useful addition in the detection of estrus.

To our knowledge, the present study is the first investigation on the changes of MIR spectra of bovine milk on the days before and on the day of estrus. Wavenumbers 1612 to 1678 cm^−1^ and 3059 to 3661 cm^−1^, which belong to the water absorption regions, showed larger variation than the other wavenumbers. The current study showed that 95 of 175 wavenumbers of the water absorption regions were significantly affected by the days in relation to estrus. Wang et al. [21] showed that wavenumbers 3466 to 3543 cm^−1^ of the water absorption region were significantly affected by the lactation stage and the DGAT1 polymorphism. Du et al. [22] indicated that wavenumbers 3040 to 3090 cm^−1^ were significantly affected by parity and lactation stage, and wavenumbers 3553 to 3657 cm^−1^ were significantly influenced by lactation stage. The wavenumbers of the water absorption regions are usually excluded when setting up prediction models [23]. However, the present study and previous studies [21,22] propose that some wavenumbers of the water absorption regions may contain important information, and should be completely or partially considered when using infrared spectral data.

The days in relation to estrus showed significant effects on numerous infrared wavenumbers, except for the water absorption regions. The changes in milk composition during estrus confirmed in the present study as well as a previous study [8] are reflected by the milk infrared wavenumbers. For the first region (wavenumbers from 925 to 1608 cm^−1^), significant changes in wavenumbers between 925 and 952 cm^−1^, between 991 and 1481 cm^−1^, and between 1500 and 1574 cm^−1^ were observed. This region is called the “fingerprint region”, referring to several peaks of absorbance, such as the C–O, C–C, C=C, C–H, N–O, C–N, amide II, and amide III bands. These chemical bonds are common in milk components such as fat, protein, lactose, carbohydrates, and organic acids [21,24,25,26]. Especially, we found extremely significant changes in some of the wavenumbers. For example, wavenumbers between 1203 and 1284 cm^−1^ were highly affected by the days in relation to estrus, which might be explained as the amide III band of the casein absorption peak is around wavenumber 1250 cm^−1^ [21].

In the third region, spanning wavenumbers from 1682 to 3055 cm^−1^, the days in relation to estrus had significant effects on most of the wavenumbers between 1682 and 2280 cm^−1^, and between 2472 to 3055 cm^−1^, which are the center for “fat A” and “fat B” [25,26]. These wavenumbers are associated with carboxylic acid, an ester C=O bond, alkyl C–H stretching, C–N bonds, and N–H bonds, which is abundant in fat [21,24,26].

Figure 3 also shows significant effects of the days in relation to estrus on most of the wavenumbers between 3665 and 5011 cm^−1^ (the last region). Wavenumbers from 4033 to 4350 cm^−1^ can be attributed to combination bands of C–H, which is abundant in fatty acids. Wavenumbers 4500 to 5000 cm^−1^ can be attributed to vibrations of the N–H and C=O groups of proteins [21]. These might explain the significant effects of days in relation to estrus on these wavenumbers.

## 5. Possible Use of Milk MIR Spectra in the Reproductive Management

MIR spectra are usually used to predict the milk composition by considering the spectral data as predictors and the reference value of a specific milk component to be predicted as the response variable [27]. Moreover, an increasing number of studies have reported a good capability of milk MIR to predict some animal characteristics, such as methane emissions [28], feed intake [29], and lameness [30].

Besides the use of milk spectra data in the above aspects, it is also proposed for reproductive management, such as estrus detection and pregnancy diagnosis. In fact, there are already some studies on the application of milk MIR spectra on pregnancy diagnosis. Lainé et al. [27] firstly reported that milk mid-infrared spectra, especially the region from wavenumbers 968 to 1577 cm^−1^, were directly affected by the pregnancy stage. Toledo-Alvarado et al. [31] subsequently developed models with spectral wavelengths as predictors to predict pregnancy status, and their results demonstrated that the spectrum has a low but positive predictive ability. Delhez et al. [32] also showed that milk MIR spectral data were not sufficient to detect the pregnancy status of dairy cows at earlier stages. However, the models developed using data recorded after 150 d of pregnancy showed a promising prediction accuracy. Besides, Ho et al. [33] indicated that milk MIR spectroscopy together with other on-farm data could be used to classify cows of good and poor likelihood of conception at the first insemination with reasonably good accuracy. Further analyses are warranted to more deeply explore the potential use of milk MIR spectra in improving the predictive accuracy of pregnancy status.

It is well documented that milk composition alters with the occurrence of estrus [8,9,10]. Importantly, the present study indicated that the days in relation to estrus had a significant effect on many infrared wavenumbers. Wang et al. [21] reported that the total phenotypic variant of wavenumbers could be explained by herd to an extent. In our previous study [22], we also showed that the herd had a significant effect on 1060 wavenumbers. In the present study, milk samples were collected only from one farm. Therefore, in future, milk samples from numerous farms need to be collected. Besides, samples from cows with spontaneous estrus also should be included to conduct a more comprehensive analysis of estrus. The logical next step will be the use of the milk MIR spectra as a support tool for diagnoses of cows in estrus or, even better, those approaching estrus, subsequently guiding their timely insemination.

## 6. Conclusions

This study showed that the composition of fat, protein, urea, TS, and SnF in milk significantly changed on the days before and on the day of estrus, whereas lactose showed no significant difference. With regard to the milk mid-infrared spectrum, some of the wavenumbers in the water absorption regions were significantly influenced by the days in relation to estrus. This suggested that these wavenumbers might contain important information. The days in relation to estrus also significantly affected numerous wavenumbers in the region containing many absorbance peaks of important chemical bonds, which indicated that the effect of estrus could be observed in the milk composition through the analysis of the MIR spectrum. Further analyses are needed to explore the potential of MIR spectra to discriminate between cows approaching versus not approaching estrus.

## Figures and Tables

**Table 1:** Descriptive statistics of the milk components.

Trait	Mean	SD	CV	Min	Max
Fat (%)	4.06	1.04	25.62	1.50	7.74
Protein (%)	3.68	0.51	13.86	2.37	5.36
Lactose (%)	5.21	0.26	4.99	2.14	5.72
Urea (mg/100 g)	18.81	5.86	31.15	5.80	33.90
TS (%)	13.81	1.34	9.70	7.78	17.60
SnF (%)	9.62	0.57	5.93	6.07	11.43

SD: standard deviation; CV: coefficient of variation; TS: total solid; SnF: solid not fat.

## Data Availability

The data that support the findings of this study are available from the corresponding author upon reasonable request.

## References

[1] Palmer M.A., Olmos G., Boyle L.A., Mee J.F. (2010). Estrus detection and estrus characteristics in housed and pastured Holstein–Friesian cows. Theriogenology.

[2] Reith S., Hoy S. (2018). Review: Behavioral signs of estrus and the potential of fully automated systems for detection of estrus in dairy cattle. Animals.

[3] Saint-Dizier M., Chastant-Maillard S. (2012). Towards an Automated Detection of Oestrus in Dairy Cattle. Reprod. Domest. Anim..

[4] Talukder S., Kerrisk K., Ingenhoff L., Thomson P., Garcia S., Celi P. (2014). Infrared technology for estrus detection and as a predictor of time of ovulation in dairy cows in a pasture-based system. Theriogenology.

[5] Leroy C., Walton J., Leblanc S. (2018). Estrous detection intensity and accuracy and optimal timing of insemination with automated activity monitors for dairy cows. J. Dairy Sci..

[6] Mayo L.M., Silvia W.J., Ray D.L., Jones B.W., Stone A.E., Tsai I.C., Clark J.D., Bewley J.M., Heersche G. (2019). Automated estrous detection using multiple commercial precision dairy monitoring technologies in synchronized dairy cows. J. Dairy Sci..

[7] Foroutan A., Guo A.C., Vazquez-Fresno R., Lipfert M., Zhang L., Zheng J., Badran H., Budinski Z., Mandal R., Ametaj B.N. (2019). Chemical Composition of Commercial Cow’s Milk. J. Agric. Food Chem..

[8] Alvarado H.T., Vazquez A.I., Campos G.D.L., Tempelman R.J., Gabai G., Cecchinato A., Bittante G. (2018). Changes in milk characteristics and fatty acid profile during the estrous cycle in dairy cows. J. Dairy Sci..

[9] Zebari H.M., Rutter S.M., Bleach E.C. (2019). Fatty acid profile of milk for determining reproductive status in lactating Holstein Friesian cows. Anim. Reprod. Sci..

[10] Zhao C., Bai Y., Fu S., Wu L., Xia C., Xu C. (2021). Comparison of Metabolic Alterations in Serum and Milk Whey Between Inactive Ovaries and Estrus Dairy Cows. Front. Vet. Sci..

[11] Etzion Y., Linker R., Cogan U., Shmulevich I. (2004). Determination of Protein Concentration in Raw Milk by Mid-Infrared Fourier Transform Infrared/Attenuated Total Reflectance Spectroscopy. J. Dairy Sci..

[12] De Marchi M., Toffanin V., Cassandro M., Penasa M. (2014). Invited review: Mid-infrared spectroscopy as phenotyping tool for milk traits. J. Dairy Sci..

[13] Soyeurt H., Bastin C., Colinet F.G., Arnould V.M.-R., Berry D.P., Wall E., Dehareng F., Nguyen H.N., Dardenne P., Schefers J. (2012). Mid-infrared prediction of lactoferrin content in bovine milk: Potential indicator of mastitis. Animals.

[14] Ferrand-Calmels M., Palhière I., Brochard M., Leray O., Astruc J., Aurel M., Barbey S., Bouvier F., Brunschwig P., Caillat H. (2014). Prediction of fatty acid profiles in cow, ewe, and goat milk by mid-infrared spectrometry. J. Dairy Sci..

[15] McDermott A., Visentin G., De Marchi M., Berry D., Fenelon M., O’Connor P., Kenny O., McParland S. (2016). Prediction of individual milk proteins including free amino acids in bovine milk using mid-infrared spectroscopy and their correlations with milk processing characteristics. J. Dairy Sci..

[16] Grelet C., Bastin C., Gelé M., Davière J.-B., Johan M., Werner A., Reding R., Pierna J.F., Colinet F., Dardenne P. (2016). Development of Fourier transform mid-infrared calibrations to predict acetone, β-hydroxybutyrate, and citrate contents in bovine milk through a European dairy network. J. Dairy Sci..

[17] Malacarne M., Visentin G., Summer A., Cassandro M., Penasa M., Bolzoni G., Zanardi G., De Marchi M. (2018). Investigation on the effectiveness of mid-infrared spectroscopy to predict detailed mineral composition of bulk milk. J. Dairy Res..

[18] Franzoi M., Niero G., Visentin G., Penasa M., Cassandro M., De Marchi M. (2019). Variation of Detailed Protein Composition of Cow Milk Predicted from a Large Database of Mid-Infrared Spectra. Animals.

[19] Moroni P., Pisoni G., Savoini G., Van Lier E., Acuña S., Damián J., Meikle A. (2007). Influence of Estrus of Dairy Goats on Somatic Cell Count, Milk Traits, and Sex Steroid Receptors in the Mammary Gland. J. Dairy Sci..

[20] Akdag F., Cadirci O., Siriken B. (2010). Effect of Estrus on Milk Yield and Composition in Jersey Cows. Bulg. J. Agric. Sci..

[21] Wang Q., Hulzebosch A., Bovenhuis H. (2016). Genetic and environmental variation in bovine milk infrared spectra. J. Dairy Sci..

[22] Du C., Nan L., Yan L., Bu Q., Ren X., Zhang Z., Sabek A., Zhang S. (2020). Genetic Analysis of Milk Production Traits and Mid-Infrared Spectra in Chinese Holstein Population. Animals.

[23] De Marchi M., Fagan C., O’Donnell C., Cecchinato A., Zotto R.D., Cassandro M., Penasa M., Bittante G. (2009). Prediction of coagulation properties, titratable acidity, and pH of bovine milk using mid-infrared spectroscopy. J. Dairy Sci..

[24] Soyeurt H., Misztal I., Gengler N. (2010). Genetic variability of milk components based on mid-infrared spectral data. J. Dairy Sci..

[25] Bittante G., Cecchinato A. (2013). Genetic analysis of the Fourier-transform infrared spectra of bovine milk with emphasis on individual wavelengths related to specific chemical bonds. J. Dairy Sci..

[26] Zaalberg R., Shetty N., Janss L., Buitenhuis A. (2019). Genetic analysis of Fourier transform infrared milk spectra in Danish Holstein and Danish Jersey. J. Dairy Sci..

[27] Lainé A., Bastin C., Grelet C., Hammami H., Colinet F., Dale L., Gillon A., Vandenplas J., Dehareng F., Gengler N. (2017). Assessing the effect of pregnancy stage on milk composition of dairy cows using mid-infrared spectra. J. Dairy Sci..

[28] van Gastelen S., Dijkstra J. (2016). Prediction of methane emission from lactating dairy cows using milk fatty acids and mid-infrared spectroscopy. J. Sci. Food Agric..

[29] Dórea J., Rosa G., Weld K., Armentano L. (2018). Mining data from milk infrared spectroscopy to improve feed intake predictions in lactating dairy cows. J. Dairy Sci..

[30] Bonfatti V., Ho P., Pryce J. (2020). Usefulness of milk mid-infrared spectroscopy for predicting lameness score in dairy cows. J. Dairy Sci..

[31] Alvarado H.T., Vazquez A.I., Campos G.D.L., Tempelman R.J., Bittante G., Cecchinato A. (2018). Diagnosing pregnancy status using infrared spectra and milk composition in dairy cows. J. Dairy Sci..

[32] Delhez P., Ho P., Gengler N., Soyeurt H., Pryce J. (2020). Diagnosing the pregnancy status of dairy cows: How useful is milk mid-infrared spectroscopy?. J. Dairy Sci..

[33] Ho P., Bonfatti V., Luke T., Pryce J. (2019). Classifying the fertility of dairy cows using milk mid-infrared spectroscopy. J. Dairy Sci..

